# An age-group analysis on the efficacy of chemotherapy in older adult patients with metastatic biliary tract cancer: a Japanese cancer registry cohort study

**DOI:** 10.1186/s12876-023-02898-x

**Published:** 2023-08-01

**Authors:** Takeru Hirao, Kenji Ikezawa, Toshitaka Morishima, Kazuma Daiku, Yusuke Seiki, Ko Watsuji, Yasuharu Kawamoto, Sena Higashi, Makiko Urabe, Yugo Kai, Ryoji Takada, Takuo Yamai, Kaori Mukai, Tasuku Nakabori, Hiroyuki Uehara, Isao Miyashiro, Kazuyoshi Ohkawa

**Affiliations:** 1 Department of Hepatobiliary and Pancreatic Oncology, Osaka International Cancer Institute, 3-1-69 Otemae, Chuo-ku, Osaka, 541-8567 Japan; 2 Cancer Control Center, Osaka International Cancer Institute, Osaka, Japan

**Keywords:** Older adult patients, Biliary tract cancer, Chemotherapy, Propensity score matching, Age-group analysis

## Abstract

**Background:**

The effectiveness of chemotherapy in older adult patients with biliary tract cancer (BTC) remains to be established, despite the fact that the majority of patients diagnosed with BTC tend to be aged ≥ 70 years. In this study, we used three databases to examine the effectiveness of chemotherapy in a large patient population aged ≥ 70 years with metastatic BTC.

**Methods:**

Using a large Japanese database that combined three data sources (Osaka Cancer Registry, Japan’s Diagnosis Procedure Combination, the hospital-based cancer registry database), we extracted the data from patients pathologically diagnosed with metastatic BTC, between January 1, 2013, and December 31, 2015, in 30 designated cancer care hospitals (DCCHs). A cohort of patients with comparable backgrounds was identified using propensity score matching. The log-rank test was used to examine how chemotherapy affected overall survival (OS).

**Results:**

Among 2,622 registered patients with BTC in 30 DCCHs, 207 older adult patients aged > 70 years with metastatic BTC were selected. Chemotherapy significantly improved the prognosis of older adult patients, according to propensity score matching (chemotherapy, 6.4 months vs. best supportive care, 1.8 months, P value < 0.001). The number of patients receiving chemotherapy tends to decrease with age. Gemcitabine plus cisplatin (GC) and gemcitabine plus S-1 (oral fluoropyrimidine) (GS) combination therapy were frequently performed in the chemotherapy group for patients under 80 years of age (70–74 years, 61.7%; 75–79 years, 62.8%). In contrast, monotherapy including GEM and S-1 was more frequently performed in age groups over 80 years (80–84 years, 56.2%; 85–89 years, 77.7%; ≥90 years, 100%). In the chemotherapy group among older adult patients aged < 85 years, the median OS was significantly longer according to age-group analysis of the 5-year age range following propensity score matching.

**Conclusions:**

In older adult patients with metastatic BTC who received chemotherapy, prolonged survival was observed. Chemotherapy may be a viable option for patients with metastatic BTC who are aged < 85 years.

**Supplementary Information:**

The online version contains supplementary material available at 10.1186/s12876-023-02898-x.

## Background

Biliary tract cancer (BTC) includes cholangiocarcinoma (intrahepatic and extrahepatic), gallbladder cancer, and carcinoma of the ampulla of Vater [1–3]. There were 22,159 estimated new cases of BTC in Japan, accounting for 2.2% of all malignant tumors, and BTC was the sixth leading cause, accounting for approximately 3.7% of all cancer deaths in 2019 [4]. The susceptibility age is 75–84 years, and approximately 80% of patients are over 70 years of age, making this disease common among older adults. Despite advances in diagnostic techniques and treatments such as chemotherapy, BTC remains one of the worst prognoses of all digestive cancers because most patients present with advanced disease at first diagnosis [5–8].

The standard systemic palliative chemotherapies for the first-line treatment of patients with advanced biliary tract cancer in Japan are gemcitabine plus cisplatin (GC), gemcitabine plus S-1 (oral fluoropyrimidine) (GS), or gemcitabine plus cisplatin plus S-1 (GCS) [9]. In 2009, Valle et al. reported the results of a phase III trial of gemcitabine monotherapy vs. GC for advanced BTC and showed that GC was significantly superior to gemcitabine monotherapy in terms of overall survival (OS) [10]. A comparative study using a similar regimen conducted in Japan showed that superior benefits were obtained with GC, which has been widely used as the global standard for palliative chemotherapy [11]. Subsequently, a phase III study was conducted in Japan to verify the non-inferiority of GS to GC [12, 13]. Also, a phase III study was conducted to test the superiority of GCS over GC, and the superiority of GCS was demonstrated [14].

Although these previous prospective randomized controlled studies enrolled patients aged > 70 years, there have been limited data regarding the efficacy of these regimens in older adult patients [15]. In this study, we aimed to investigate the effectiveness of chemotherapy in a large patient population aged ≥ 70 years with metastatic BTC using three databases. Propensity score-matched analyses were performed to adjust for patient background. We also performed age-group analyses to examine the age at which chemotherapy was effective.

## Methods

### Data sources

In this study, we combined three data sources to obtain a large, consolidated database of clinical information and mortality. Data from patients diagnosed with metastatic BTC between 2013 and 2015 were analyzed.

The Osaka Cancer Registry was our first data source and contained population-based information on cancer diagnoses and outcomes among the residents of Osaka Prefecture, Japan. As of May 2019, death certificates and official resident registers were used to verify the date of death or the last follow-up [16–21].

The second data source was administrative information generated by the Diagnosis Procedure Combination (DPC) Per Diem Payment System in Japan, a widely used hospital administrative data source in Japan [22]. It prescribes reimbursements from insurers to acute care hospitals who provide healthcare goods and services. Clinical summaries, such as unique hospital identifiers, admission dates, diagnoses, patient characteristics (including body height, weight, and Barthel Index score), medications (drug name and date of drug administration), and pre-existing comorbidities on admission can be extracted from these data. The DPC data were obtained from 30 designated cancer care hospitals (DCCHs) in Osaka Prefecture, which were certified as hospitals that can provide high-quality cancer care by The Japanese Ministry of Health, Labor and Welfare and Osaka Prefecture Government. The International Classification of Diseases, Tenth Revision (ICD-10) codes were used to document diagnoses and comorbidities.

The third data source was the hospital-based cancer registry database, which contains the following tumor characteristics: topographical and morphological codes of the International Classification of Diseases for Oncology, Third Edition (ICD-O-3), demographics (birth date and sex), tumor node-metastasis classification, stage according to the Seventh Edition of the Union for International Cancer Control staging system, date of cancer diagnosis, and diagnosis stage (surgery, radiation, or chemotherapy).

Hospital-assigned identification numbers for each hospital were used as linkage keys to connect the three data sources. Approximately half of the new cancer cases diagnosed in Osaka Prefecture during the research period were included in this database.

### Patients

**Fig. 1:**
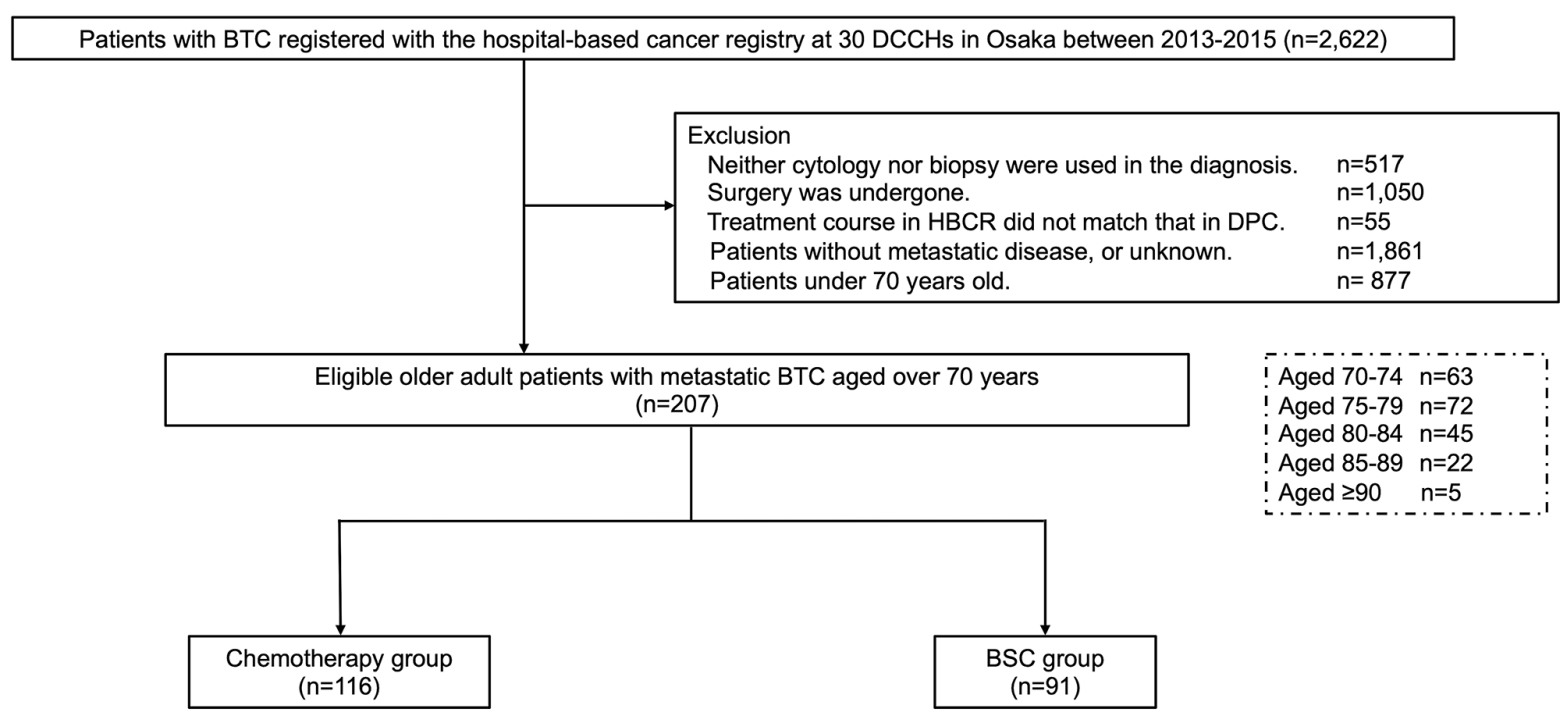
Overview of patient selection and stratification by use of chemotherapy Abbreviations: BTC, biliary tract cancer; DCCH, Designated Cancer Care hospital; HBCR, hospital-based cancer registry; DPC, Diagnosis Procedure Combination; BSC, best supportive care

At the DCCHs in Osaka Prefecture between January 1, 2013, and December 31, 2015, we first identified 2,622 patients who had been diagnosed with BTC. Patients with BTC in this study met the two criteria listed below: (a) patients whose ICD-O-3 topographical codes were C22.1 or C23.9 or C24.0 or C24.1, and (b) patients whose ICD-O-3 morphological codes were neoplasm, malignant (8000/3); carcinoma in situ, not otherwise specified (NOS) (8010/2); carcinoma, NOS (8010/3); adenocarcinoma in situ, NOS (8140/2); adenocarcinoma, NOS (8140/3); cholangiocarcinoma (8160/3); tubular adenocarcinoma, NOS (8211/3); papillary adenocarcinoma, NOS (8260/3); noninfiltrating intraductal papillary adenocarcinoma (8503/2); and adenosquamous carcinoma (8560/3). Subsequently, to ensure the exclusive collection of data from older adult patients with pathologically-proven metastatic BTC, we excluded the following patient data: (a) BTC was not cytopathologically proven (n = 517); (b) the initial selected treatment was surgery or information on surgery was unavailable (n = 1,050); (c) information on chemotherapy as initial treatment obtained by DPC was not concordant with that in hospital-based cancer registry (n = 55); (d) patients without metastatic disease, or unknown (n = 1,861); and (e) patients younger than 70 (n = 877) (Fig. 1).

### Variables and clinical outcomes

We extracted age at diagnosis along with other factors such as sex, body mass index (BMI) (< 19, ≥ 19), tumor location, functional status, and pre-existing comorbidities, as baseline patient characteristics. The tumor location was divided into gallbladder and non-gallbladder, as gallbladder cancer has a poorer survival outcome in BTC [11, 12, 23]. Using the Barthel Index score we evaluated the functional status, which includes 10 items to gauge performance in activities of daily living (ADL). Higher overall scores indicate better levels of independence [21, 24]. The patients were divided into two groups according to their Barthel Index score (0–94, 95–100). The updated Charlson Comorbidity Index (CCI) score was used to evaluate comorbidities. To assess the comorbidities of patients, excluding metastases, we utilized the Quan adaptation of the CCI based on ICD-10 codes [25, 26]. According to previously published processes, patients were classified into two groups: those without comorbidities (CCI score:0) and those with comorbidities (CCI score:1) [27].

Using the DPC data, we collected information on the chemotherapy regimens. The following four regimens were frequently used throughout the study period:1) GC, 2) GS, 3) gemcitabine monotherapy (GEM), and 4) monotherapy with S-1. The OS was defined as the period between the date of diagnosis and the date of the last follow-up or death from any cause.

### Statistical analysis

Categorical variables are expressed as numbers and percentages, and continuous variables are expressed as means and standard deviations. Student’s t-test or chi-square test, as applicable, was used to assess all variables. The Kaplan-Meier method of survival analysis was used, and the log-rank test was used to assess differences. Propensity score matching was used to identify patients with similar baseline characteristics. Briefly, a 1:1 matching protocol without replacement (greedy-matching algorithm) was used for matching with a caliper width equal to 0.2 of the standard deviation of the logit of the propensity score. Before and after matching, to assess pre- and post-match balances, standardized differences were determined for each baseline covariate. Small imbalances were indicated when the normalized differences for each covariate were less than 0.1 [28]. The Mantel-Haenszel test was used for paired comparisons in the matched cohort.

The percentage of missing values was 0-13.5% for the six variables. In total, 28 (13.5%) of 207 records were incomplete. In this study, missing values existed only for categorical variables (BMI) (13.5%), Barthel Index (7.7%). We allocated each missing value to a category with a larger number of cases to reduce the effect of missing values.

EZR (Saitama Medical Center, Jichi Medical University, Saitama, Japan), a graphical interface for R, and the R Commander software package for Windows were used to perform all statistical analyses (version 1.54) [29]. P values less than 0.05 were considered statistically significant.

## Results

Among the 2,622 registered patients with BTC in 30 DCCHs, 207 patients aged > 70 years with metastatic BTC were included in this study (Fig. 1). ICD-O-3 morphological codes of these patients were as follows; adenocarcinoma NOS (8140/3), n = 128; cholangiocarcinoma (8160/3), n = 53; tubular adenocarcinoma NOS (8211/3), n = 13; malignant (8000/3), n = 8; carcinoma NOS (8010/3), n = 4; and papillary adenocarcinoma NOS (8260/3), n = 1.

**Fig. 2:**
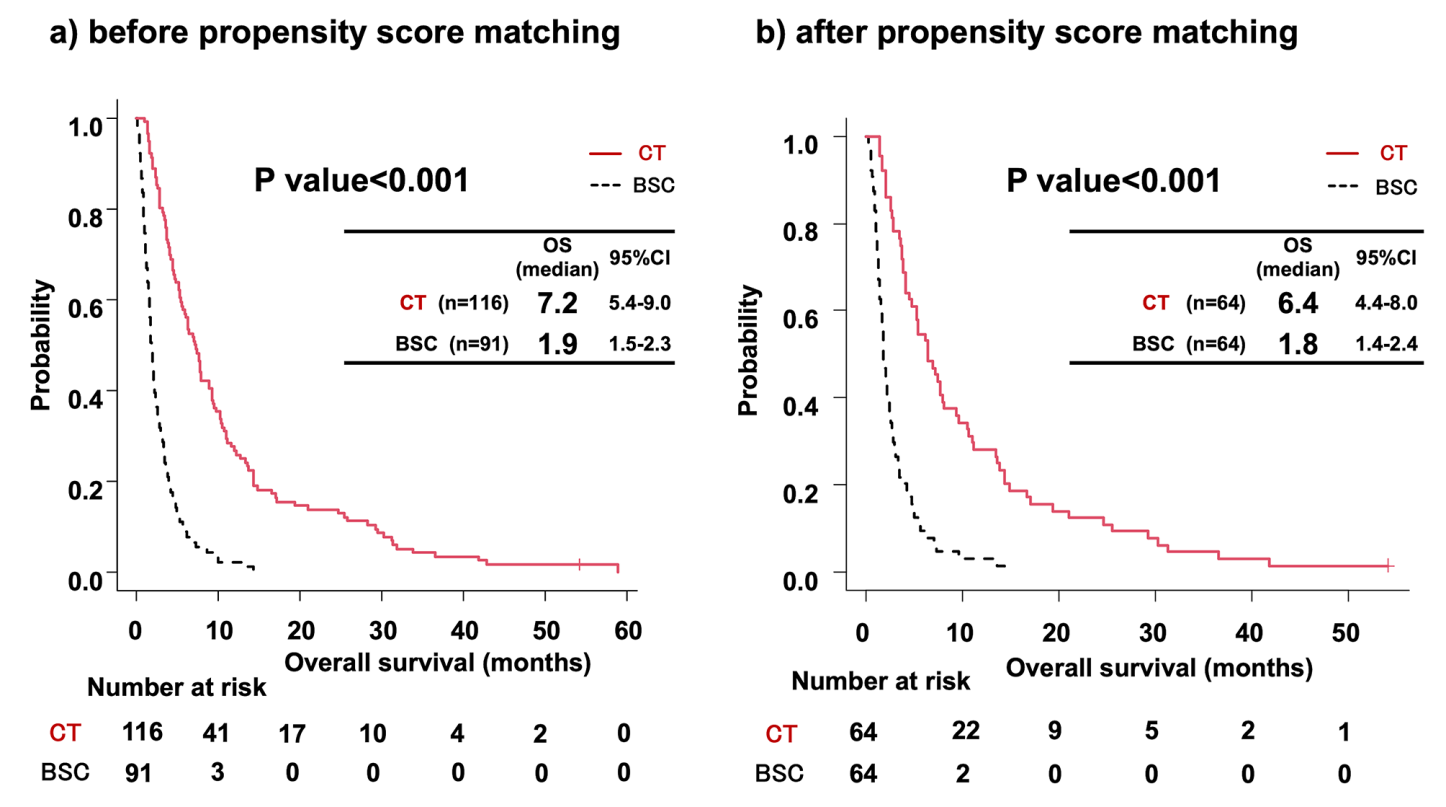
Overall survival comparison between the BSC and chemotherapy group. **(a)** Analysis before propensity score matching and **(b)** Analysis after propensity score matching Abbreviations: CT, chemotherapy; BSC, best supportive care; OS, overall survival

First, we compared older adult patients with metastatic BTC who underwent chemotherapy (chemotherapy group) with those who received the best supportive care (BSC group) to clarify the impact of chemotherapy on OS. Table 1 shows a comparison of patient characteristics between the two groups. Before performing the propensity score-matched analyses, we found significant differences in age and Barthel Index scores between the two groups. Non-gallbladder cancer included intrahepatic cholangiocarcinoma (n = 61), extrahepatic cholangiocarcinoma (n = 68), and carcinoma of the ampulla of Vater (n = 6). After propensity score-matched analyses, 64 patients in each group were included, and there were no significant differences in any of the variables. The standardized differences in age, sex, tumor location, and Barthel Index were less than 0.1 after propensity score-matched analyses. In both analyses (before and after propensity score-matched analyses), the chemotherapy group exhibited a significantly more favorable OS than the BSC group (before propensity score-matched analyses, 7.2 months vs. 1.9 months [P value < 0.001]; after propensity score-matched analyses, 6.4 months vs. 1.8 months [P value < 0.001]) (Fig. 2). In the subgroup analysis according to tumor location, the chemotherapy group demonstrated a significantly longer OS than the BSC group in patients with gallbladder cancer, intrahepatic cholangiocarcinoma, and extrahepatic cholangiocarcinoma (Supplementary Fig. 1^1^).Next, we performed age-group analyses by dividing patients aged > 70 years with metastatic BTC into five age groups:70–74, 75–79, 80–84, 85–89, and ≥ 90 years. The frequency of patients receiving chemotherapy decreased with age (Table 2). In the chemotherapy group, combination therapy, including GC and GS, was frequently performed in the age groups < 80 years (70–74 years, 61.7%; 75–79 years, 62.8%). Among the age groups < 80 years, OS was 8.4 months in GC, 4.2 months in GS, 5.9 months in GEM, and 7.3 months in S-1. In contrast, monotherapy including GEM and S-1 was more frequently performed over 80 years age groups (80–84 years, 56.2%; 85–89 years, 77.7%; ≥90 years, 100%). Among the age groups > 80 years age groups, OS was 7.9 months in GC, 6.9 months in GEM, and 4.4 months in S-1.

**Table 1:** Baseline characteristics among older adult patients with metastatic BTC before and after propensity score matching

	Original Data Set					Matched Data Set			
Variables	Chemotherapy (n = 116)	BSC (n = 91)	Sdiff	*P value		Chemotherapy (n = 64)	BSC (n = 64)	Sdiff	†P value
Age, mean (SD)	76.5 (4.93)	79.7 (5.49)	0.618	< 0.001		78.2 (5.01)	78.1 (4.70)	0.032	0.856
Sex, n (%)									
Male	63 (54.3)	50 (54.9)	0.013	> 0.99		36 (56.2)	35 (54.7)	0.031	> 0.99
Female	53 (45.7)	41 (45.1)				28 (43.8)	29 (45.3)		
Body mass index, n (%)									
<19	14 (12.1)	15 (16.5)	0.126	0.422		8 (12.5)	11 (17.2)	0.132	0.62
≥19	102 (87.9)	76 (83.5)				56 (87.5)	53 (82.8)		
Tumor Location, n (%)									
Gallbladder	45 (38.8)	27 (29.7)	0.193	0.188		24 (37.5)	21 (32.8)	0.098	0.711
Non-Gallbladder	71 (61.2)	64 (70.3)				40 (62.5)	43 (67.2)		
Barthel Index, n(%)									
≥95	105 (90.5)	60 (65.9)	0.624	< 0.001		53 (82.8)	53 (82.8)	< 0.001	> 0.99
≤94	11 (9.5)	31 (34.1)				11 (17.2)	11 (17.2)		
CCI, n (%)									
0	68 (58.6)	55 (60.4)	0.037	0.887		38 (59.4)	33 (51.6)	0.158	0.477
≥1	48 (41.4)	36 (39.6)				26 (40.6)	31 (48.4)		

BSC, best supportive care; SD, standard deviations; CCI, Charlson Comorbidity Index; Sdiff, standardized difference.

*P value for Student t-test or Chi-square test; †P value for Mantel-Haenszel test.

**Table 2:** Distribution of chemotherapy regimens among the five age groups

Age group	1) 70–74 (n = 63)	2) 75–79 (n = 72)	3) 80–84 (n = 45)	4) 85–89 (n = 22)	4) ≥ 90 (n = 5)
	n (%)	n (%)	n (%)	n (%)	n (%)
Chemotherapy					
Yes	47 (74.6)	43 (59.7)	16 (35.6)	9 (40.9)	1 (20.0)
No	16 (25.4)	29 (40.3)	29 (64.4)	13 (59.1)	4 (80.0)
1st line					
GC	28 (59.6)	26 (60.5)	7 (43.8)	2 (22.2)	0 (0.0)
GS	1 (2.1)	1 (2.3)	0 (0.0)	0 (0.0)	0 (0.0)
GEM	13 (27.7)	11 (25.6)	6 (37.5)	4 (44.4)	1 (100)
S-1	5 (10.6)	5 (11.6)	3 (18.7)	3 (33.3)	0 (0.0)

GC, gemcitabine plus cisplatin; GS, gemcitabine plus S-1

GEM, gemcitabine; S-1, oral fluoropyrimidine

**Fig. 3:**
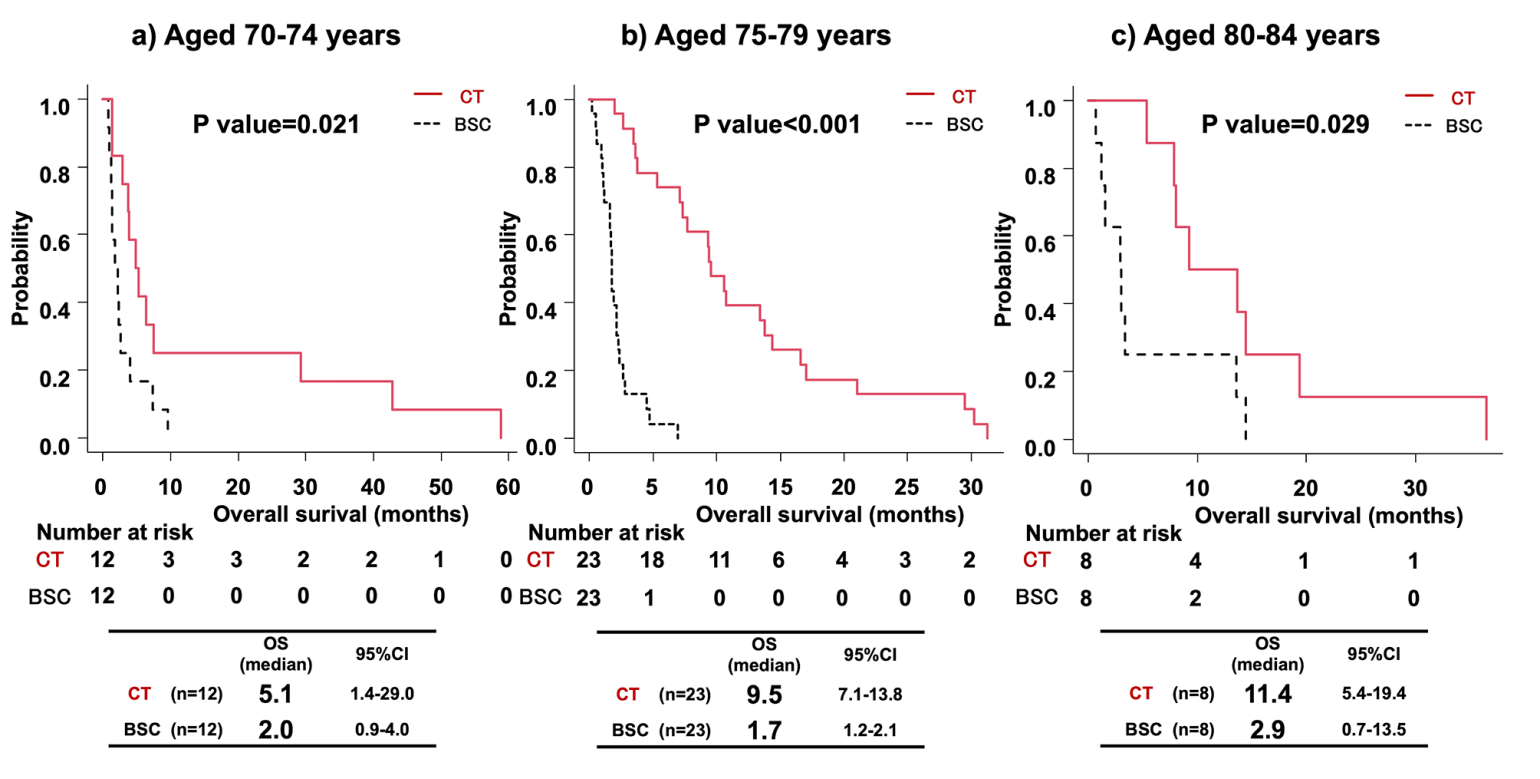
Overall survival comparison in age groups between BSC and chemotherapy groups after propensity score matching. **(a)** Analysis of patients aged 70–74, **(b)** analysis of patients aged 75–79 and **(c)** analysis of patients aged 80–84. Abbreviations: CI, confidence interval; OS, overall survival

To examine the age at which chemotherapy is effective, we compared the chemotherapy group with the BSC group in 70–74-, 75–79-, and 80–84-year-old patients before and after propensity score-matched analyses. As shown in Supplementary Tables 1–3, there were no significant differences in the patient backgrounds for all variables after propensity score matching. The chemotherapy group demonstrated a significantly longer OS than the BSC group among patients aged 70–74, 75–79, and 80–84 years both before propensity score-matched analyses (data not shown) and after propensity score-matched analyses (Fig. 3).

## Discussion

The target age for cancer research in older adult patients is not clearly defined, and study definitions of this age vary. As nearly 90% of people with clinical indications of aging are over 70, 70, or 75 has been chosen as the cutoff age for older adult patients in different clinical trials [30]. In clinical practice, functional decline, cognitive dysfunction, and frailty around the age of 70 years and older are causes of concern; thus, treatment decisions should be made cautiously. In this study, to understand the outcomes of older adult patients with metastatic BTC, we collected data on patients who were aged ≥ 70 years.

The number of studies investigating the efficacy and safety of chemotherapy in older adults with advanced BTC has gradually increased in recent years. Patients aged < 80 years with a diagnosis of hepatopancreaticobiliary malignancy were examined in a retrospective study in Poland, of the 1,421 patients, 10% were over 80, and of those over 80, 36% had BTC. Patients aged < 80 years and those aged > 80 years who were receiving palliative systemic anti-cancer therapy had median OS of 10.07 months (95% confidence interval (CI):8.89–11.08) and 10.10 months (95% CI:6.30–12.30), respectively; P value = 0.41 [31]. When considered physically fit enough, older adult patients experienced an OS advantage on par with younger patients. Also, a Japanese multicenter retrospective study compared patients with BTC < 75 years (N = 94) and ≥ 75 years (N = 309) treated with palliative chemotherapy from 2006 to 2009 and reported that OS of older adult patients receiving palliative chemotherapy was comparable with that of younger adult patients (median OS:11.5 months vs. 10.4 months (hazard ratio 1.14 (95% CI:0.89–1.45), P value = 0.31) [32]. Similarly, in this study, after correcting for variations in patient background using propensity score-matched analyses, older adult patients with pathologically-proven metastatic BTC who underwent chemotherapy exhibited a considerably better prognosis than those who chose BSC. These findings, which were derived from a large real database that took into account functional status and pre-existing comorbidities, may encourage physicians to consider chemotherapy as a treatment option for older adults with metastatic BTC in clinical settings.

Although GC and GS are the most frequently prescribed standard therapies for patients with metastatic BTC, due to the perceptions of potential increased toxicity and increased presence of comorbidities, physicians in general clinical practice may be reluctant to administer combination therapy rather than monotherapy in older adult patients. In this study using real-world data, among older adult patients with metastatic BTC who underwent chemotherapy, 56.1% underwent combination therapy (GC or GS) and 43.1% underwent monotherapy (GEM or S-1). Combination therapy was frequently performed in the age-group < 80 years (70–74 years, 61.7%; 75–79 years, 62.8%), and the number of patients who underwent monotherapy was higher in the age-group > 80 years. Previously, an integrated analysis of 13 clinical trials showed that combination therapy is equally effective at age 70 and older [33]. Moreover, a subgroup analysis of JCOG1113 (randomized Phase III study of GC versus GS in advanced biliary tract cancer) was performed recently focusing on older adult patients with BTC [34]. They reported that survival benefits with gemcitabine-based combination chemotherapy were similar between older adult patients and non-older adult patients. Furthermore, the frequency of all-grade adverse events was also similar between older adult patients and non-older adult patients. The survival of older adult patients with BTC receiving systemic chemotherapy is comparable to that of younger individuals for whom data are available. In the intricate decision-making processes involved in identifying older adult patients who may derive greater benefits from potentially more toxic combination therapies, it is imperative to employ comprehensive geriatric assessment tools [35]. These validated tools may assist in determining the health status of older adult patients, their medical and social requirements, and their individual preferences, rather than relying solely on chronological age, to choose appropriate regimens for older adult patients with metastatic BTC.

This study also examined the oldest age at which older adult patients with metastatic BTC benefited from chemotherapy. Little information is available on the age at which chemotherapy is effective. An extensive study from the Netherlands, which did not include patients with BTC patients but patients with pancreatic cancer (PC), found that median OS was considerably better in the chemotherapy group as compared to the untreated group in patients with PC aged ≥80 years. Patient backgrounds were not adjusted for in this analysis [36]. In contrast, after accounting for variations in patient backgrounds using propensity-score matched analyses, Daiku et al. found that chemotherapy was a significant determinant of better outcomes in patients aged < 85 years with metastatic PC [19]. Similarly, age-group analyses following propensity score matching for metastatic BTC in the current study showed that chemotherapy improved the survival of patients aged < 85 years. After propensity score matching, chemotherapy proved to be a significant factor for better prognosis in patients aged 70–74 years, 75–79 years, and 80–84 years. This finding suggests that patients aged < 85 years may be promising candidates for chemotherapy. Meanwhile, because the number of patients aged ≥ 85 years was limited, it was difficult to determine the benefit of chemotherapy for these patients. Recently, immunotherapeutic approaches have been actively investigated [2, 37–40]. The success of the TOPAZ-1 trial (GC plus durvalumab) is promising, and recently, the efficacy of GC plus pembrolizumab compared with GC was reported in KEYNOTE-966 [41, 42]. Thus, the use of immune checkpoint inhibitors (ICIs) with chemotherapy would evidently expand [43, 44]. Our understanding of the clinical activities and toxicity profiles of ICIs in older adult patients is limited by their underrepresentation in clinical trials [45, 46]. New toxicities, including immune-related toxicities, are presumed to appear, and the selection of treatment targets in older adult patients may become more important. Further studies with more extensive data and age group analyses are required to determine the benefits of chemotherapy in older patients.

This study had several limitations. First, the results of this retrospective study were from a single Asian country and may lack international standardization. Second, several important variables affecting the prognosis of older adult patients, such as laboratory data, tumor markers [47], performance status [48–50], and pretreatment geriatric assessment were not available in the database. Although adjusting all the possible factors associated with the prognosis, such as biliary drainage [51–53], was challenging, important factors including age, sex, body mass index, tumor location, functional status, and pre-existing comorbidities, were included in propensity score matching. Additionally, based on other database investigations, the Barthel Index was substituted for performance status [21, 24, 54, 55]. Third, because this study was a retrospective study, patients who were eligible for chemotherapy could potentially exhibit favorable Barthel index scores. Propensity score matching was performed for this cohort; thus, the efficacy of chemotherapy could be limited in older adult patients with good performance. Fourth, as a characteristic of the data sources used in this study, we did not obtain sufficient information on the changes in the quality of life and symptoms over time, or treatment courses during and after first-line chemotherapy in individual cases. Thus, the examination of items, such as the frequency of adverse effects, quality of life changes, and levels of discomfort during and after first-line chemotherapy administration should be conducted in future studies to provide additional justification for recommending palliative chemotherapy in older adult patients.

## Conclusions

In this study, propensity score-matched analyses using a Japanese cancer registry indicated that chemotherapy provided a survival benefit in older adult patients (aged > 70 years) with metastatic BTC. Patients with metastatic BTC aged < 85 years could be promising candidates for chemotherapy.

## Electronic supplementary material

Below is the link to the electronic supplementary material.

Supplementary Material 1

Supplementary Material 2

Supplementary Material 3

## Data Availability

The data that support the findings of this study are available upon reasonable request from the corresponding author. The data are not publicly available due to privacy and ethical restrictions.

## Abbreviations

BMI: Body mass index
BTC: Biliary tract cancer
CCI: Charlson Comorbidity Index
CI: Confidence interval
DCCH: Designated cancer care hospitals
DPC: Diagnosis Procedure Combination
GC: Gemcitabine plus cisplatin
GCS: Gemcitabine plus cisplatin plus S-1
GEM: Gemcitabine
GS: Gemcitabine plus S-1
HBCR: Hospital-based cancer registry
HPB: Hepatopancreaticobiliary
OS: Overall survival
PC: Pancreatic cancer
