# Supplementary material for: An age-group analysis on the efficacy of chemotherapy in older adult patients with metastatic biliary tract cancer: a Japanese cancer registry cohort study
Source: BMC Gastroenterol. 2023 Aug 1;23:263. doi: 10.1186/s12876-023-02898-x (PMC10391780; doi:10.1186/s12876-023-02898-x)
Supplement: Supplementary file 1 — Supplementary Material 1 [file 12876_2023_2898_MOESM1_ESM.pptx]

## Slide 1
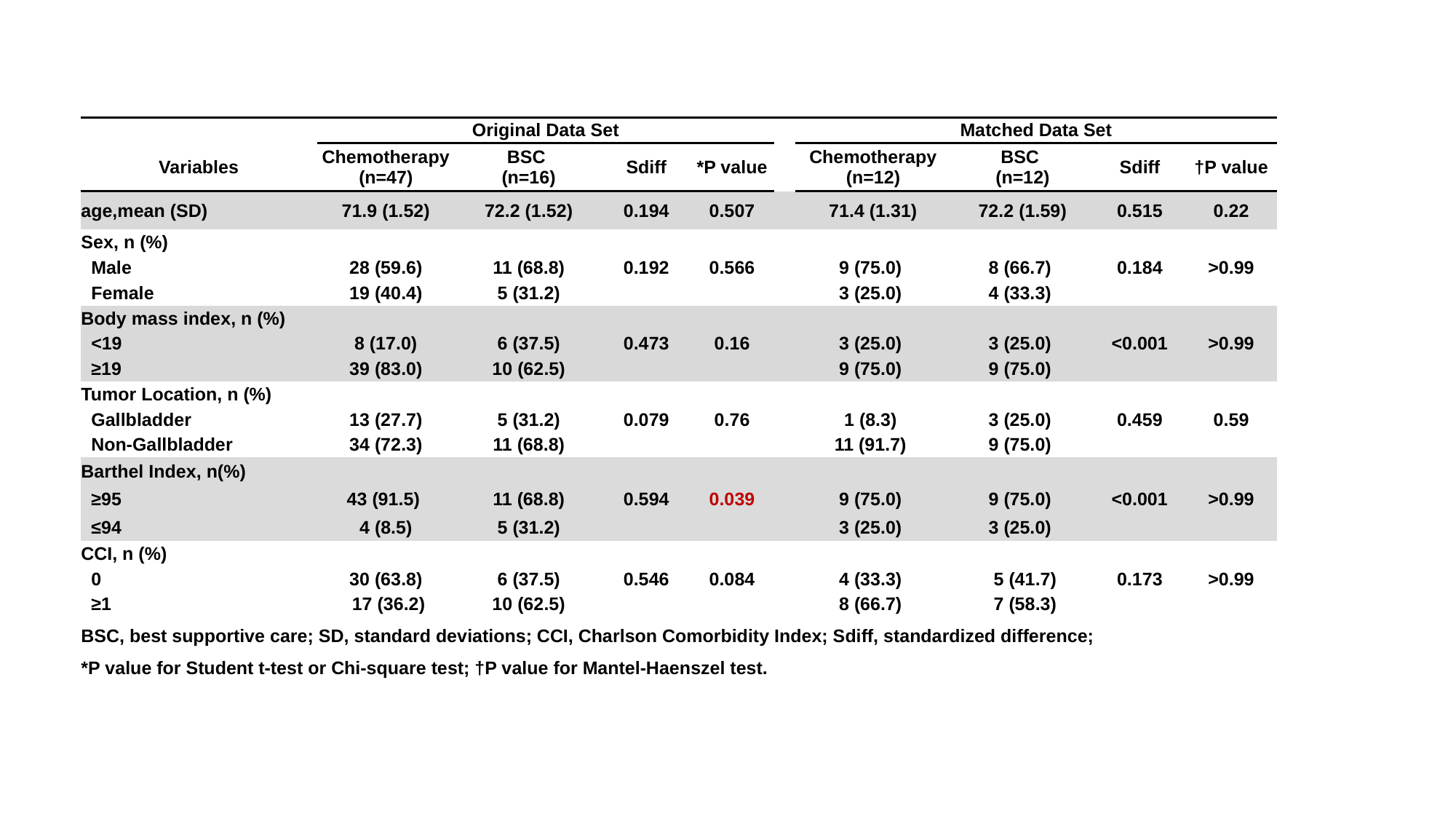

| | Original Data Set | | | | | Matched Data Set | | | |
| --- | --- | --- | --- | --- | --- | --- | --- | --- | --- |
| Variables | Chemotherapy (n=47) | BSC (n=16) | Sdiff | \*P value | | Chemotherapy (n=12) | BSC (n=12) | Sdiff | †P value |
| age,mean (SD) | 71.9 (1.52) | 72.2 (1.52) | 0.194 | 0.507 | | 71.4 (1.31) | 72.2 (1.59) | 0.515 | 0.22 |
| Sex, n (%) | | | | | | | | | |
| Male | 28 (59.6) | 11 (68.8) | 0.192 | 0.566 | | 9 (75.0) | 8 (66.7) | 0.184 | >0.99 |
| Female | 19 (40.4) | 5 (31.2) | | | | 3 (25.0) | 4 (33.3) | | |
| Body mass index, n (%) | | | | | | | | | |
| <19 | 8 (17.0) | 6 (37.5) | 0.473 | 0.16 | | 3 (25.0) | 3 (25.0) | <0.001 | >0.99 |
| ≥19 | 39 (83.0) | 10 (62.5) | | | | 9 (75.0) | 9 (75.0) | | |
| Tumor Location, n (%) | | | | | | | | | |
| Gallbladder | 13 (27.7) | 5 (31.2) | 0.079 | 0.76 | | 1 (8.3) | 3 (25.0) | 0.459 | 0.59 |
| Non-Gallbladder | 34 (72.3) | 11 (68.8) | | | | 11 (91.7) | 9 (75.0) | | |
| Barthel Index, n(%) | | | | | | | | | |
| ≥95 | 43 (91.5) | 11 (68.8) | 0.594 | 0.039 | | 9 (75.0) | 9 (75.0) | <0.001 | >0.99 |
| ≤94 | 4 (8.5) | 5 (31.2) | | | | 3 (25.0) | 3 (25.0) | | |
| CCI, n (%) | | | | | | | | | |
| 0 | 30 (63.8) | 6 (37.5) | 0.546 | 0.084 | | 4 (33.3) | 5 (41.7) | 0.173 | >0.99 |
| ≥1 | 17 (36.2) | 10 (62.5) | | | | 8 (66.7) | 7 (58.3) | | |
| BSC, best supportive care; SD, standard deviations; CCI, Charlson Comorbidity Index; Sdiff, standardized difference; | | | | | | | | | |
| \*P value for Student t-test or Chi-square test; †P value for Mantel-Haenszel test. | | | | | | | | | |

## Slide 2
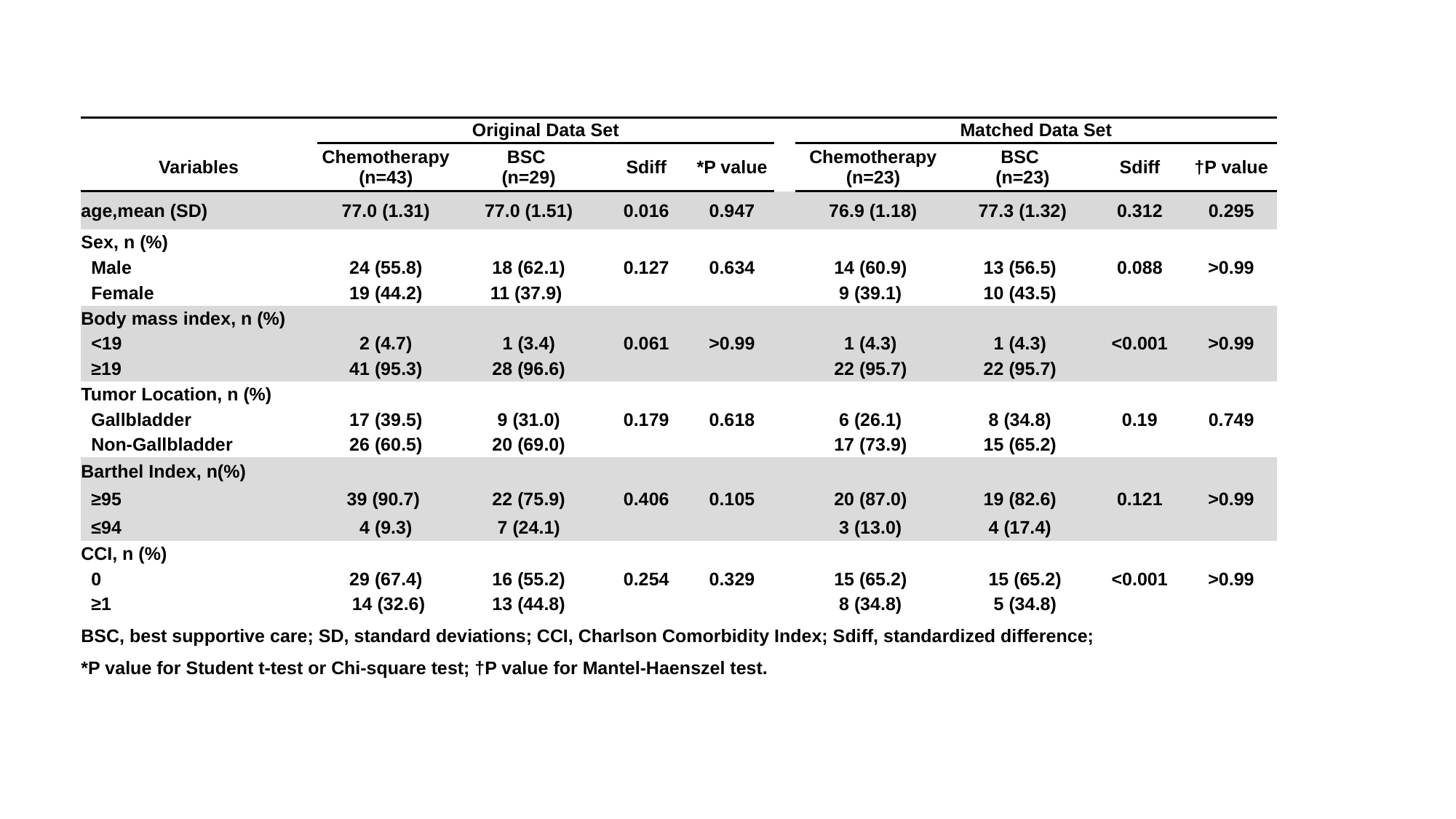

| | Original Data Set | | | | | Matched Data Set | | | |
| --- | --- | --- | --- | --- | --- | --- | --- | --- | --- |
| Variables | Chemotherapy (n=43) | BSC (n=29) | Sdiff | \*P value | | Chemotherapy (n=23) | BSC (n=23) | Sdiff | †P value |
| age,mean (SD) | 77.0 (1.31) | 77.0 (1.51) | 0.016 | 0.947 | | 76.9 (1.18) | 77.3 (1.32) | 0.312 | 0.295 |
| Sex, n (%) | | | | | | | | | |
| Male | 24 (55.8) | 18 (62.1) | 0.127 | 0.634 | | 14 (60.9) | 13 (56.5) | 0.088 | >0.99 |
| Female | 19 (44.2) | 11 (37.9) | | | | 9 (39.1) | 10 (43.5) | | |
| Body mass index, n (%) | | | | | | | | | |
| <19 | 2 (4.7) | 1 (3.4) | 0.061 | >0.99 | | 1 (4.3) | 1 (4.3) | <0.001 | >0.99 |
| ≥19 | 41 (95.3) | 28 (96.6) | | | | 22 (95.7) | 22 (95.7) | | |
| Tumor Location, n (%) | | | | | | | | | |
| Gallbladder | 17 (39.5) | 9 (31.0) | 0.179 | 0.618 | | 6 (26.1) | 8 (34.8) | 0.19 | 0.749 |
| Non-Gallbladder | 26 (60.5) | 20 (69.0) | | | | 17 (73.9) | 15 (65.2) | | |
| Barthel Index, n(%) | | | | | | | | | |
| ≥95 | 39 (90.7) | 22 (75.9) | 0.406 | 0.105 | | 20 (87.0) | 19 (82.6) | 0.121 | >0.99 |
| ≤94 | 4 (9.3) | 7 (24.1) | | | | 3 (13.0) | 4 (17.4) | | |
| CCI, n (%) | | | | | | | | | |
| 0 | 29 (67.4) | 16 (55.2) | 0.254 | 0.329 | | 15 (65.2) | 15 (65.2) | <0.001 | >0.99 |
| ≥1 | 14 (32.6) | 13 (44.8) | | | | 8 (34.8) | 5 (34.8) | | |
| BSC, best supportive care; SD, standard deviations; CCI, Charlson Comorbidity Index; Sdiff, standardized difference; | | | | | | | | | |
| \*P value for Student t-test or Chi-square test; †P value for Mantel-Haenszel test. | | | | | | | | | |

## Slide 3
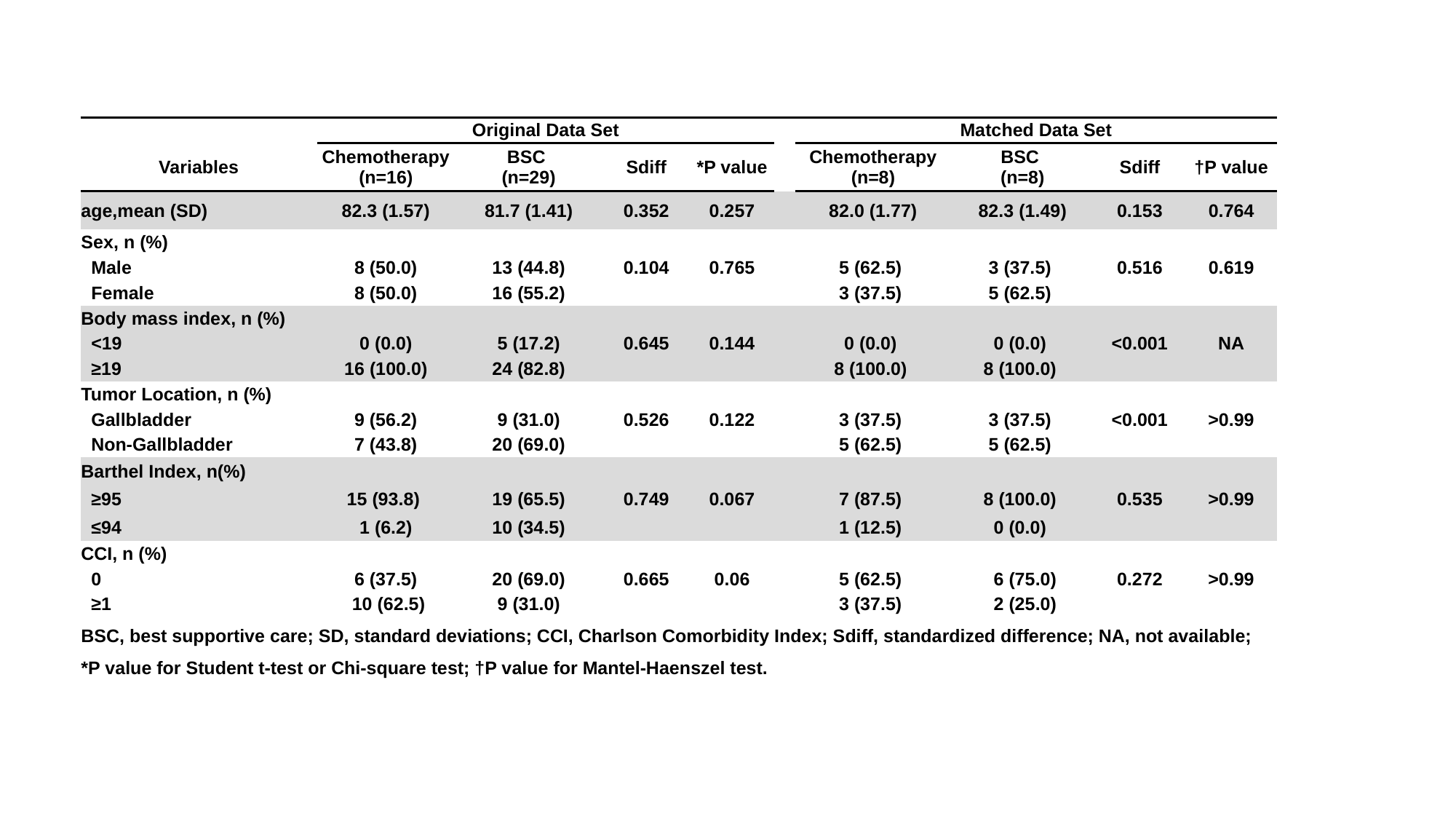

| | Original Data Set | | | | | Matched Data Set | | | |
| --- | --- | --- | --- | --- | --- | --- | --- | --- | --- |
| Variables | Chemotherapy (n=16) | BSC (n=29) | Sdiff | \*P value | | Chemotherapy (n=8) | BSC (n=8) | Sdiff | †P value |
| age,mean (SD) | 82.3 (1.57) | 81.7 (1.41) | 0.352 | 0.257 | | 82.0 (1.77) | 82.3 (1.49) | 0.153 | 0.764 |
| Sex, n (%) | | | | | | | | | |
| Male | 8 (50.0) | 13 (44.8) | 0.104 | 0.765 | | 5 (62.5) | 3 (37.5) | 0.516 | 0.619 |
| Female | 8 (50.0) | 16 (55.2) | | | | 3 (37.5) | 5 (62.5) | | |
| Body mass index, n (%) | | | | | | | | | |
| <19 | 0 (0.0) | 5 (17.2) | 0.645 | 0.144 | | 0 (0.0) | 0 (0.0) | <0.001 | NA |
| ≥19 | 16 (100.0) | 24 (82.8) | | | | 8 (100.0) | 8 (100.0) | | |
| Tumor Location, n (%) | | | | | | | | | |
| Gallbladder | 9 (56.2) | 9 (31.0) | 0.526 | 0.122 | | 3 (37.5) | 3 (37.5) | <0.001 | >0.99 |
| Non-Gallbladder | 7 (43.8) | 20 (69.0) | | | | 5 (62.5) | 5 (62.5) | | |
| Barthel Index, n(%) | | | | | | | | | |
| ≥95 | 15 (93.8) | 19 (65.5) | 0.749 | 0.067 | | 7 (87.5) | 8 (100.0) | 0.535 | >0.99 |
| ≤94 | 1 (6.2) | 10 (34.5) | | | | 1 (12.5) | 0 (0.0) | | |
| CCI, n (%) | | | | | | | | | |
| 0 | 6 (37.5) | 20 (69.0) | 0.665 | 0.06 | | 5 (62.5) | 6 (75.0) | 0.272 | >0.99 |
| ≥1 | 10 (62.5) | 9 (31.0) | | | | 3 (37.5) | 2 (25.0) | | |
| BSC, best supportive care; SD, standard deviations; CCI, Charlson Comorbidity Index; Sdiff, standardized difference; NA, not available; | | | | | | | | | |
| \*P value for Student t-test or Chi-square test; †P value for Mantel-Haenszel test. | | | | | | | | | |
