# Supplementary figures and images for: An age-group analysis on the efficacy of chemotherapy in older adult patients with metastatic biliary tract cancer: a Japanese cancer registry cohort study
Source: BMC Gastroenterol. 2023 Aug 1;23:263. doi: 10.1186/s12876-023-02898-x (PMC10391780; doi:10.1186/s12876-023-02898-x)

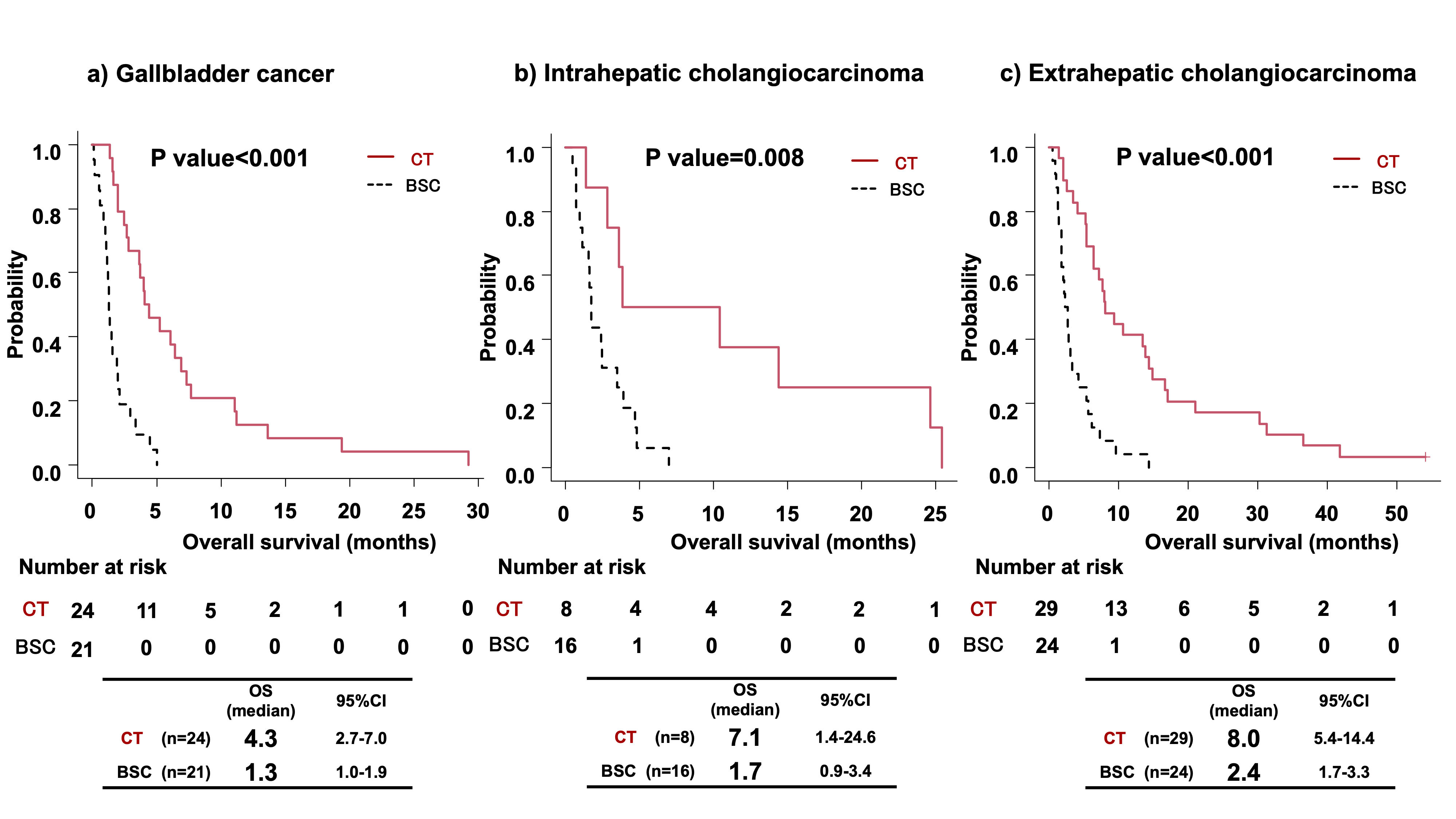

Supplement: Supplementary file 2 — Supplementary Material 2 [file 12876_2023_2898_MOESM2_ESM.tiff]
