# Supplementary material for: An age-group analysis on the efficacy of chemotherapy in older adult patients with metastatic biliary tract cancer: a Japanese cancer registry cohort study
Source: BMC Gastroenterol. 2023 Aug 1;23:263. doi: 10.1186/s12876-023-02898-x (PMC10391780; doi:10.1186/s12876-023-02898-x)
Supplement: Supplementary file 3 — Supplementary Material 3 [file 12876_2023_2898_MOESM3_ESM.docx]

**Supplementary figure legend:**

**Fig. 1** Analysis of the overall survival comparison between the best supportive care (BSC) and chemotherapy group after propensity score matching, according to the tumor location.

1. Gallbladder cancer. b) Intrahepatic cholangiocarcinoma. C) Extrahepatic cholangiocarcinoma.
